# Geospatial Mapping and Meteorological Flood Risk Assessment: A Global Research Trend Analysis

**DOI:** 10.1007/s00267-024-02059-0

**Published:** 2024-10-12

**Authors:** Phila Sibandze, Ahmed Mukalazi Kalumba, Amal H. Aljaddani, Leocadia Zhou, Gbenga Abayomi Afuye

**Affiliations:** 1 Department of GIS and Remote Sensing, University of Fort Hare, P/Bag X1314, Alice, 5700 South Africa; 2 Department of Geography and Environmental Science, University of Fort Hare, Private Bag X1314, Alice, 5700 Eastern Cape Province South Africa; 3 Geospatial Application, Climate Change and Environmental Sustainability Lab–GACCES, University of Fort Hare, Alice, 5700 Eastern Cape Province South Africa; 4 Department of Physical Sciences, College of Science, University of Jeddah, Jeddah, Saudi Arabia; 5 Risk and Vulnerability Science Centre, University of Fort Hare, Alice, 5700 South Africa

**Keywords:** Flood risk assessment, Flood risk, Flood control, Geospatial flood, Meteorological flood

## Abstract

Flooding is a global threat causing significant economic and environmental damage, necessitating a policy response and collaborative strategy. This study assessed global research trends and advances in geospatial and meteorological flood risk assessment (G_MFRA), considering the ongoing debate on flood risk management and adaptation strategies. A total of 1872 original articles were downloaded in BibTex format using the Web of Science (WOS) and Scopus databases to retrieve G_MFRA studies published from 1985 to 2023. The annual growth rate of 15.48% implies that the field of G_MFRA has been increasing over time during the study period. The analysis of global trends in flood risk research and practice highlights the key themes, methodologies, and emerging directions. There exists a notable gap in data and methodologies for flood risk assessment studies between developed and developing countries, particularly in Africa and South America, highlighting the urgency of coordinated research efforts and cohesive policy actions. The challenges identified in the body of extant literature include technical expertise, complex communication networks, and resource constraints associated with the application gaps of the study methodologies. This study advocates for a holistic research approach to flood disaster management through ecosystem-based adaptation that underpins the Sustainable Development Goals to develop innovative flood techniques and models with the potential to influence global decision-making in the G_MFRA domain. Addressing these global challenges requires a networked partnership between the research community, institutions, and countries.

## Introduction

The significant rise in flood risks in recent decades remains a major cause for concern due to its devastating effects on communities, residents, and the natural environment, encompassing vital aquatic ecosystem services. (Leal Filho et al. 2021; Wing et al. 2022). Climate change and rapid population growth threaten humanity, the environment, and their long-term sustainability (Mavrouli et al. 2022; Afuye et al. 2022). This has led to a surge in the number of global disasters, with hydrological events (159; 48.2%) being among the most severe in comparison to meteorological disasters (106; 32.1%), climatological (33; 10%) and geophysical disasters (32; 9.7%) accounting for the total largest share of natural disasters (Tabish and Syed,^ 2015^; Guha-Sapir et al. 2017; Mpanyaro et al. 2024). The assessment report on global disasters indicated that floods accounted for 52% of catastrophic events in 2021, with 23% of the worldwide population (1.3 billion) exposed to flood depths of 0.15 m, where a third were from China (395 million) and India (390 million) (EM-DAT 2022a; Rentschler et al. 2022). According to the Intergovernmental Panel on Climate Change (IPCC) reports, flood risks and their associated societal impacts are projected to increase the magnitude of global warming further (IPCC 2022a; Asadnabizadeh 2022). This implies that many regions may experience more severe flood risks with considerable shifts in temperature, with particular emphasis on coastal cities, considering anticipated increases in sea level, storm surges, and coastal flooding (IPCC 2022b). Climate change is predicted to increase the risk of flooding in the next 30 years, putting over half of the population at risk, while developed countries like the United States, Germany and the Netherlands are embroiled in the ongoing global debate (Sadiq et al. 2019; Polka 2018). Despite the significant losses and disruptions caused by rapid urbanisation, poor land use, and population increase, developed countries are less prone to flooding and inundation (Merz et al. 2021; Afuye et al. 2024a). Due to its significant societal and economic losses, this paradox provides an example of improved risk management and prevention strategies. Investment in flood protection must continue as developed nations are forced to make continuous commitments to flood mitigation and management (McDermott 2022).

The world population, scientists, and climate change advocates are concerned about the rising flooding risks, particularly in low- and middle-income nations most susceptible to inadequate response and mitigation strategies (Nguyen and Liou 2019; Bhattacharjee et al. 2021). Due to the complexities of flood risks, addressing these challenges calls for an all-encompassing collaborative approach that integrates adaptation and mitigation, considering flood risk management and environmental and socioeconomic aspects. Studies have shown that flood risks are multifaceted and result from the interplay of several factors and their underlying causes (Nguyen and Liou 2019; Hochrainer-Stigler et al. 2024). These interactions can occasionally produce trade-off outcomes and short- and long-term synergies. The damages and disruption in the changing pattern of flood occurrences through socioeconomic factors such as population density, infrastructure vulnerability, topography, urban land use, soil type and other disturbances are derived from those alterations (Douglas 2017; Nguyen and Liou 2019; Bhattacharjee et al. 2021; Ighile et al. 2022). The degree to which flood risk affects settlement areas, including urban and coastal communities, caused by natural and human-induced impacts is a global issue, receiving little attention, especially in developing nations with particular emphasis on social impact and the type of floods (Doocy et al. 2013; Tascón-González et al. 2020; Haque et al. 2020; Kunze and Strobl 2024). The effects of floods on developed and developing nations in many regions, including Africa, are highly prone to flooding because the continent has experienced the world’s highest urban and coastal city expansion (Alves et al. 2020; Angelakis et al. 2023). Many studies have focused on the physical characteristics of floods, leaving a notable gap in data and methodologies for flood risk assessment between developed and developing countries (Collet et al. 2018; Díez-Herrero and Garrote 2020; Morante-Carballo et al. 2022; Schoppa et al. 2024). This highlights the urgency of coordinated research efforts and cohesive policy actions. To address the challenges associated with flood risk, geospatial and meteorological data based on methodologies play a crucial role in developing potential solutions to the issues posed by flood disasters. These methodologies facilitate how geospatial and meteorological flood risk assessment (G_MFRA) studies have been interconnected to key trends, themes, and emerging directions. The use of G_MFRA studies has been greatly aided by these methodologies, which take context to solve complex interconnections and provide viable solutions to flood risks based on natural and socioeconomic systems (Sahani et al. 2019; Mishra et al. 2022). Understanding the gaps and appropriate solutions in the context of environmental and socioeconomic aspects of flood risk and management demands empirical knowledge and evidence-based methodologies to achieve better outcomes despite the advances in data used for G_MFRA and associated methodologies in disaster risk reduction (DRR) for both natural and socioeconomic systems. An extensive empirical assessment of its applicability to disaster risk management concerns facing humanity has not been fully explored (Dewan and Dewan 2013; Li et al. 2019; Rehman et al. 2019; Tripathi et al. 2022; Chakrabortty et al. 2023). This study is crucial because there is limited knowledge of the application gaps of the study methodologies based on geospatial and meteorological data to fully understand flood risk assessment and management, especially in developing countries.

This study used published articles to evaluate global research trends and advances in geospatial and meteorological flood risk assessment to identify key themes, gaps, methodologies, and emerging directions. Bibliometric analysis has been widely used in flood risk assessment to evaluate numerous research efforts and appropriateness in various disciplines (Estoque et al. 2019; Sharma et al. 2019; Zhu et al. 2023). However, this study evaluates multiple significant categories within G_MFRA studies. This includes the analysis of annual scientific output, the distribution of top global articles, institutional affiliations, collaboration networks, global research hotspots, trending topics and the evolution of keyword co-occurrence. This study will aid in identifying potential research gaps and dynamics on G_MFRA globally by focusing on these high-flying categories. The study aimed to enhance flood risk assessment and management studies using geospatial and meteorological data and methodologies by advancing their applicability in the field. The findings can guide future planning and research for flood disaster management and climate resilience initiatives, enhancing the development of effective strategies and aiding professionals and stakeholders in these crucial areas.

## Study Material and Methods

**Fig. 1:**
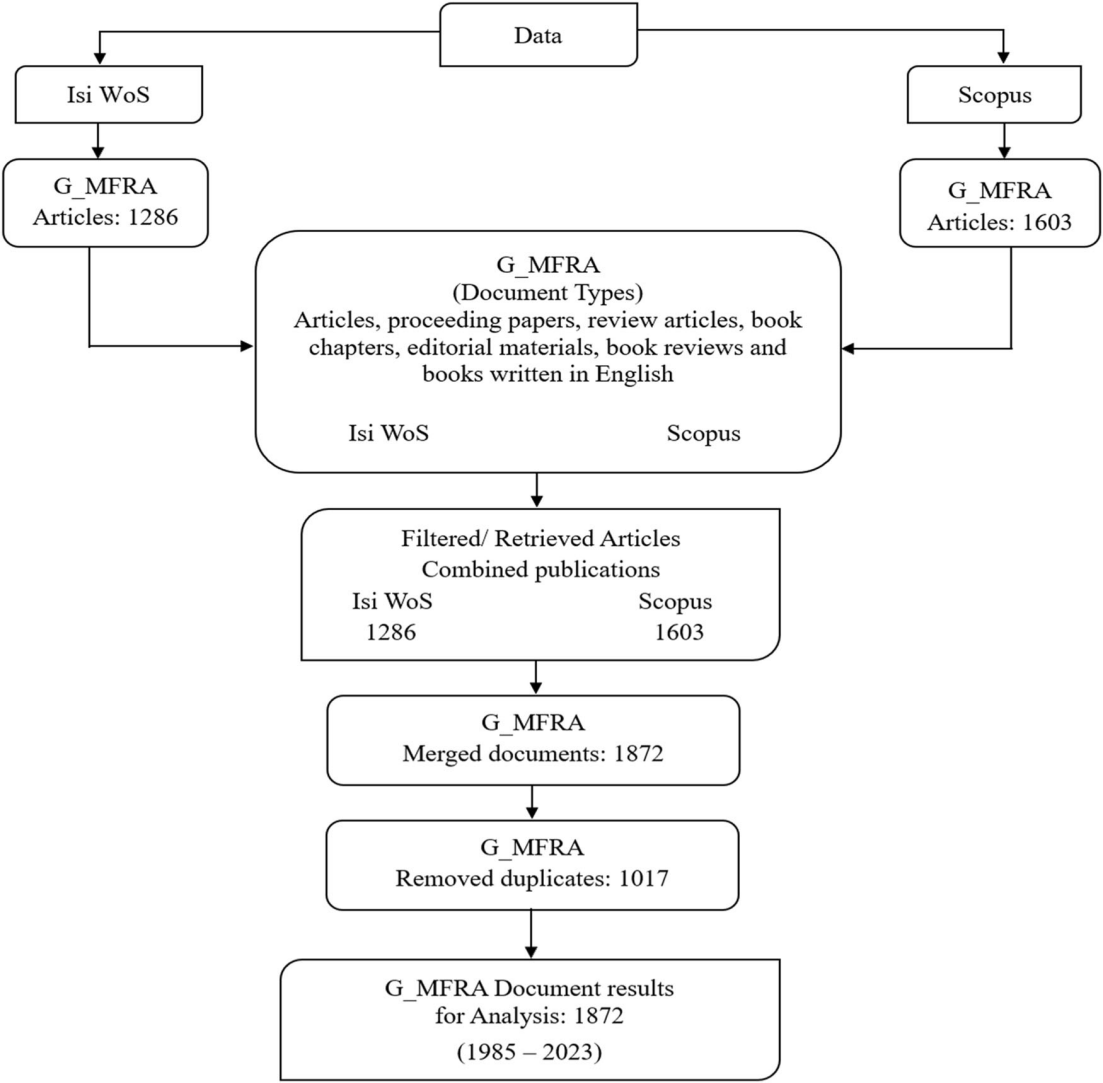
Graphical workflow showing the criteria for publication selection

On March 18, 2024, the Scopus and Isi Web of Science databases were used to retrieve English research publications on geospatial and meteorological flood risk assessment (G_MFRA) studies from the Isi Web of Science (WOS) and Scopus databases. The databases were chosen based on their reliability and research coverage, efficiency, and high-impact scientific research (Mansoori 2018; Birkle et al. 2020; Visser et al. 2021). This study used search criteria for WOS and Scopus based on these keywords: (“Geospatial and Meteorological Flood”) OR TITLE (“Flood Risk Assessment”) OR (“Spatial Flood Risk Assessment”) OR (“Meteorological Flood Risk Modelling”) AND PUBYEAR > 1985 AND PUBYEAR < 2023 in title, abstracts, and keywords (author keywords and keywords plus) of articles published between January 1985 and December 2023 (Fig. 1). Other studies have used the exact titles and topics to query the databases to ensure considerable recovery and limited loss compared to several searches (Nduku et al. 2021; Echchakoui 2020; Kasaraneni and Rosaline 2022). In addition, the retrieved published articles included articles, proceeding papers, review articles, book chapters, editorial materials, book reviews, and books.

### Study Data Processing and Validation

The study searched published original articles and reviews and screened and removed non-traceable papers and all other document categories, as presented in Fig. 1. Accordingly, data cleaning, screening, and processing were applied to remove duplicates and irrelevant documents and reduce the vocabulary dimension (Gagolewski 2011). Zotero software v6.0.7 was used to organise all retrieved published documents using the R repository’s Citation Analysis program (CITAN), which filters out phrases that are not meaningful (Aria et al. 2022; Nduku et al. 2023). Consequently, choosing these variables was due to their unique attributes and contribution to the body of knowledge (Halevi et al. 2017; Orimoloye et al. 2022).

## Results and Discussion

The analysis was based on 1872 articles from the WOS and Scopus databases, explicitly focusing on G_MFRA, as shown in the summary of the primary data retrieved in Table 1. During the study period, 4975 international authors collaborated, with a global co-authorship index of 4.915%. At the same time, 109 authors published single-authored documents that contributed to and reinforced advancements in scientific research publications on G_MFRA during the study period. The evaluated published articles were from 653 sources of journals, books, etc., with an average citation of 20.87 per document, 4.07 co-authors, and an average age of 6.68 per document in the field (Table 1). The average yearly percentage increase rate of citations per article for analysis was 15.48%. In recent decades, efforts and resources have been directed toward global research on G_MFRA to develop efficient mitigation and response strategies for flood-related disasters.

**Table 1:** Descriptive information on G_MFRA studies

Description	Results
Isi Web of Science, Scopus	2
Timespan	1985–2023
Sources (Journals, Books, etc.)	653
Documents	1872
Annual Growth Rate %	15.48
Document Average Age	6.68
Average citations per doc	20.87
References	0
*Document contents*	
Keywords Plus (ID)	6789
Author’s Keywords (DE)	4249
*AUTHORS*	
Authors	4975
Authors of single-authored docs	109
*Authors collaboration*	
Single-authored docs	148
Co-Authors per Doc	4.07
International co-authorships %	4.915
*Document types*	
Article	1256
Article Book Chapter	2
Article Conference Paper	1
Article; Book Chapter	5
Article; Data Paper	1
Article; Early Access	44
Article; Proceedings Paper	6
Article; Retracted Publication	15
Book	8
Book Chapter	68
Book Review	3
Conference Paper	324
Conference Paper Data Paper	1
Conference Review	20
Data Paper	5
Editorial Material; Book Chapter	4
Proceedings Paper	63
Review	41
Review; Early Access	4
Review; Retracted Publication	1

### Annual Scientific Output and Institutional Affiliations

**Fig. 2:**
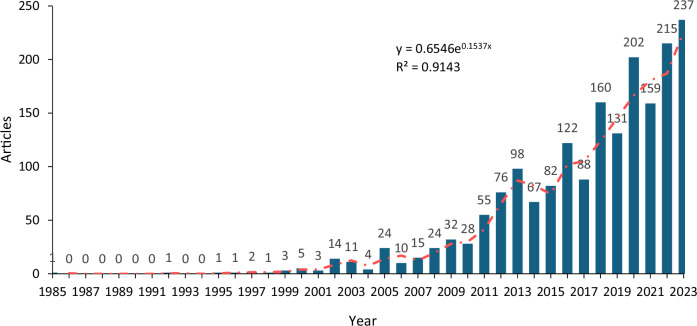
The annual scientific output on G_MFRA studies

The annual scientific output rate on G_MFRA publications revealed the research trend from a low to a yearly high production rate determined by the number of articles published between 1985 and 2023 (Fig. 2). The annual growth rate of 15.48% suggests that the field of G_MFRA has been increasing over time during the survey period. The notable gradual increase in article publication rate began in 2000 with five publications, followed by a decline of three publications between 2002 and 2004. This may be explained by the preliminary integration of sophisticated geospatial model applications in the field. Furthermore, a sharp peak in the publishing rate was observed in 2005, followed by a minor fall between 2006 and 2007. The increased trend in 2005 coincides with the observed peak in publications, particularly when the field began receiving global attention to a multidisciplinary research approach to reducing flood risk. It is worth noting that a random variation was observed between 2008 and 2023, implying that the research trend on G_MFRA studies was unstable regarding citations and publications. However, some dips were observed in 2010, 2014, 2015, 2017, 2019, and 2021; rather than maintaining the same growth rate across the period studied, research output fluctuated steadily from 2007 to 2023. The overall publication trend increased between 2006 and 2023, indicating that scholars began to engage more robustly in G_MFRA studies. This shows that this field of research began to gain traction in 2011 with 55 research articles, while in 2023, it was observed with the highest number of publications, accounting for 237 articles. Moreover, a low research output was observed, with a slight decline in 2021. The results revealed two periods of consistent publication trends, with the first period (0–1) publication between 1985 and 1998, while the second observed period shows a steady growth rate of publications between 1999 and 2023. This demonstrates how the applications of geospatial and meteorological techniques have been contributing significantly to flood disaster-related studies.

**Fig. 3:**
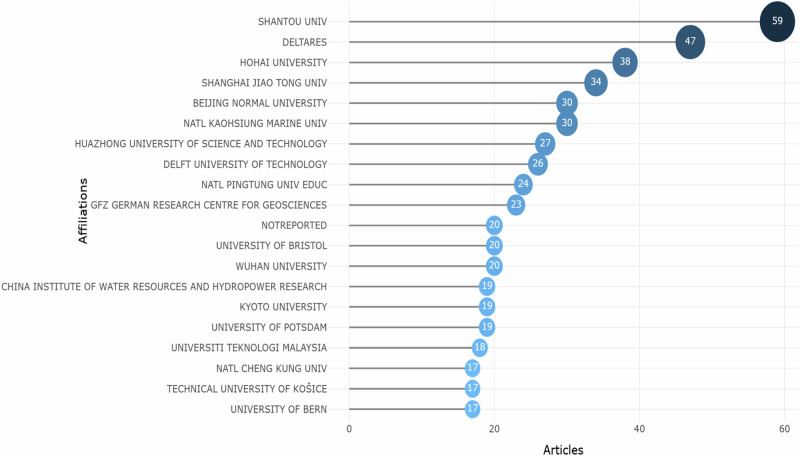
Top 20 institutional affiliations per article publications on G_MFRA studies

Figure 3 illustrates the top 20 contributors in terms of publication output from affiliated institutions within the field of G_MFRA studies. Shatou University was ranked first and closely followed by Deltares and Hohai University with (*n* = 59 articles, 3.15%), (*n* = 47, 2.51%) and (*n* = 38, 2.03%) published articles, respectively. Collaborative pathways among Chinese scholars were largely intra-national, as indicated by many articles. However, three different country affiliations were broadly significant: China, the Netherlands and Taiwan. This shows that the production of articles in the field by affiliated institutions needs to be more prominent among the top universities, suggesting that the research erudition is mainly across institutions and research centres in Asia. However, from a global perspective, the affiliations of respective countries have played a key role in shaping the structure of collaborative networks at the national and local scales. The results revealed that most of the studies on G_MFRA were from universities in the global South, while a few came from developed countries. Notably, China is significant in G_MFRA research, securing the top spots in publication output within this domain. Chinese-affiliated institutions demonstrate a prominent role in shaping the landscape of scientific research related to G_MFRA. Remarkably, Universities in Asia, particularly China, stand out among other institutions in the top 20 countries (Fig. 3), contributing to the total number of publications in the field. A study shows that China has been advancing international collaborations through several mobility funding initiatives in recent decades (Quan et al. 2019). The exceptional performance underscores China’s increasing research capacity, possibly aided by autonomous research funding initiatives (Fedderke and Goldschmidt 2015). Such development signals a notable increase in research productivity within the G_MFRA domain over the study period.

### Network Analysis of Keyword Co-occurrence and Trending Topics

**Fig. 4:**
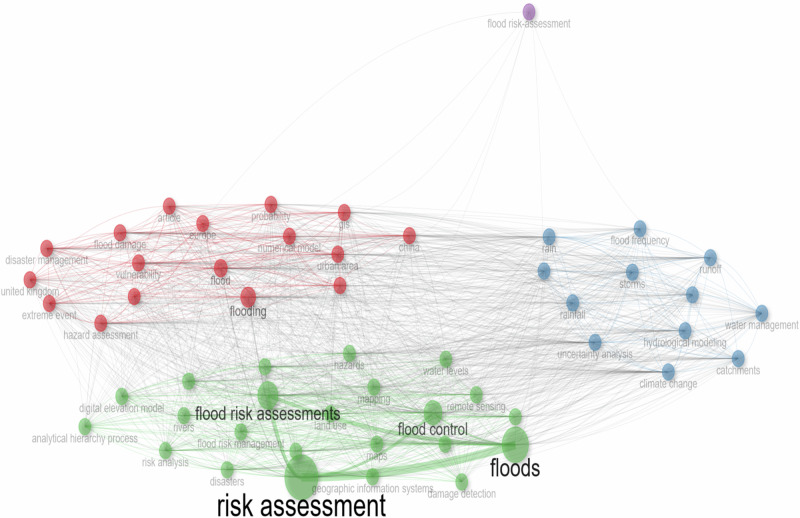
Network analysis of keyword co-occurrence in flood risk assessments research from 1985 to 2023

Figure 4 presents a visual representation of the network used to delve into the co-occurrence of author keywords, revealing the intricate landscape of research. The results show that flood risk and risk assessment are central in all keywords with a dominant appearance in the G_MFRA studies. Each circle within the intellectual network denotes the frequency of a specific keyword’s usage in the analysed niche area. Notably, flood risk assessments, risk assessment, and flood control emerge as prominent keywords within this domain, as identified through the author’s keyword search. The interconnectedness depicted in the visualisation underscores the complex relationships among these keywords, highlighting the author’s shared focus on advancing flood-related research holistically. The size of each keyword in the density and network visualisation mirrors its significance and occurrence frequency in geospatial and meteorological flood risk assessment research. The proximity between keywords suggests potential interactions over the study period. The results show significant variations in keyword co-occurrence density and network visualisation across articles, emphasising the multidimensional nature of this scientific domain (Fig. 4). These findings corroborate earlier studies by Afuye et al. (2021b) and Busayo et al. (2022). Studies have indicated that climate change and flood risk assessment have evolved into system-thinking approaches to earth observation, geoinformation science, and technology (Pollard et al. 2018; Bhunia and Shit 2022; Awah et al. 2024). This study, therefore, provides a basis for evaluating flood risks posed by natural and human-induced changes and their crippling impacts on the environment. Consequently, the network of co-occurrence keywords provides critical research hotspots for future research development and planning for climate-related flood risks to be prioritised as a crucial component of global policy interventions (IPCC 2022a; Khodadad et al. 2023).

**Fig. 5:**
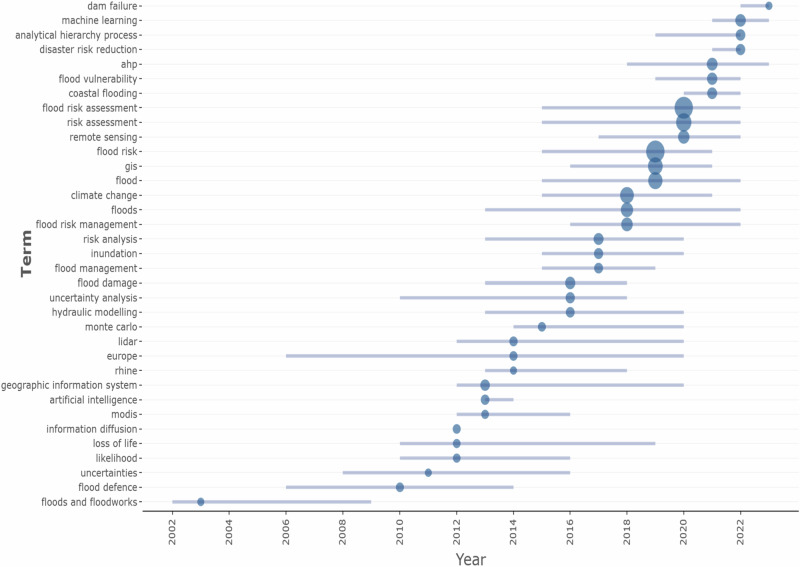
Decadal trending topics of high-frequency keywords on G_MFRA studies between 2002 and 2023

**Fig. 6:**
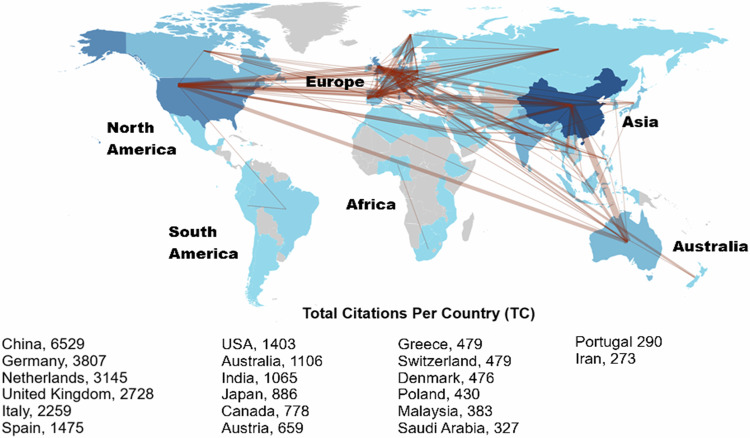
World collaboration network and distribution of top 20 total citations

Figure 5 shows the dominant keyword terms in G_MFRA between 2002 and 2023. The results identified several combinations of decadal trending topics of high-frequency keywords. Before 2002, the social effects and different types of floods were given more attention with little emphasis on study methodologies. Therefore, the time frame between 2022 and 2023 demonstrates when flood risk assessment studies began receiving global attention, particularly in 2002. The frequency of the author’s keywords on G_MFRA was compiled using a structured scheme to categorise the core trending topic of high-frequency keywords with word frequency greater than or equal to 10 selected. Therefore, thirty-five high keywords were obtained based on their occurrence field and created as decadal trending topics, as shown in Fig. 6. The results show that ‘flood risk assessment,” “risk assessment,” and “remote sensing” have more prominent nodes with significant influence in 2020. Within the last two decades, geographic information system “(GIS),” “flood risk,” and “flood” and “climate change,” “floods” and “flood risk management” also appeared to have gained dominance in 2018 and 2019 respectively. The term “machine learning,” analytical hierarchy process,” and “disaster risk reduction” have recently led to the development of system thinking approaches that feed into and build upon one another to reduce unexpected human losses and the downstream damages related to dam failure between 2022 and 2023. The integrated framework of machine learning models and analytic hierarchy processes for flood risk assessment are appropriate strategies in disaster management for reducing the effects of flood disasters (Chen et al. 2020; Pham et al. 2021). In recent decades, machine learning algorithms have been increasingly utilised to manage and predict flood risks, particularly within ecosystem-based adaptation and nature-based solutions (Pham et al. 2021). However, these approaches align with Smart/Sustainable Development Goals, offering practical solutions for mitigating flood risks. These algorithms help develop risk management strategies by providing early warnings, optimising flood control infrastructure, and guiding land-use planning to reduce flood impacts on communities and the environment (Chen et al. 2020; Ighile et al. 2022). Thus, communities must be protected from the negative consequences of flood disasters through ecosystem protection, restoration, and sustainable management (Pham et al. 2021; Busayo et al. 2022). Consequently, the dominance of these methodologies in flood risk assessment can be attributed to the increased access to free satellite data and robust cloud computing platforms such as Google Earth Engine and Amazon Web Services (Peter et al. 2020; Pandey et al. 2022; Ganjirad and Delavar 2023). Therefore, as shown in Fig. 6, these methodologies have contributed mainly to adopting geospatial and meteorological assessment of flood risk studies with an increased focus between 2002 and 2023.

### Global Research Collaboration, Distribution of Top Global Articles and the Conceptual Evolution of Author’s Keywords

Figure 6 shows the global collaboration network analysis and distribution of the top 20 citations between 1985 and 2023. This highlights the top research collaborations between countries that contributed to the field of G_MFRA. A darker shade of blue and thicker linkages between the nodes show a country’s level of dominance in the field. The grey shading indicates the regions that do not rank in the top 20 list of countries. Therefore, the darker shade of grey suggests a low level of research collaboration network from developing continents, including Africa and South America, defined by underwhelming research output due to inadequate funding for research sponsorship. This trend might have contributed to the low productivity of research in these regions (Fig. 6). The results suggest challenges encountered when conducting research in developing nations. These challenges vary from region to region, ranging from funding sources to academic research capabilities and collaborations during the survey period. Global distribution of flood occurrences shows that developed countries accounted for more flood disaster losses, suggesting a noticeably higher research collaboration on productivity than developing nations (Kundzewicz et al. 2014; Brakenridge 2016; Peng et al. 2024). Many studies have identified low research collaboration on outputs, indicating that there are few studies conducted on G_MFRA research between developed and developing continents and regions (da Silva et al. 2020; Sugam et al. 2023; Datta et al. 2023; Awah et al. 2024). Consequently, this underscores the geographical bias in research productivity, particularly in underrepresented regions, which further corroborates the findings of this study. On the other hand, the results reveal a robust collaboration network analysis between Asia, Europe, North America, and Australia. The research collaboration among these multiple-country publications (MCP) was based on geographical and institutional proximity and showed a dominant trend. Hence, we can infer that most studies on G_MFRA were sustained by articles from multiple-country publications compared to articles from single-country publications (SCP), which showed a relatively low trend. This connotes that the dominant research trend from MCP is crucial to advancing the openness of scientific research and productivity within this field of study.

Furthermore, results show the top 20 most cited countries in G_MFRA research (Fig. 6). The results show that China (*N* = 6529) ranked as the most productive country in terms of productivity based on the number of citations per country status, followed by Germany (*N* = 3807), the Netherlands (*N* = 3145), the United Kingdom (*N* = 2728), and Italy (*N* = 2259) respectively. These developed countries are the most cited in G_MFRA studies, thus revealing their robust applications in geospatial technology and meteorological models for flood risk assessment. Interestingly, developing nations like China, Poland, India, Malaysia, and Saudi Arabia made it to the top list among other developed countries. Based on the number of publications and citations per country status, no African nor South American country appeared on the top 20 lists of developed and developing countries. This draws attention to the flooding that has impacted underdeveloped nations, particularly those in Africa, with severe consequences on communities, residents, and the natural environment, including vegetation dynamics in wetland ecosystems (Ngongalah et al. 2018; Ocha 2029; Gosset et al. 2023; Afuye et al. 2024b). Flood events specifically referenced Sustainable Development Goals: SDG_11_ (Sustainable cities and communities) and SDG_13_ (climate action) were highlighted in line with G-MFRA to validate the findings presented in the study. For example, the 1121 flood catastrophe reports documented in Africa between 1985 and 2022 reveal the need for increased focus on G_MFRA research in Africa as well as other parts of the world (EM-DAT 2022a; EM-DAT 2022b; Torre Zaffaroni et al. 2023). Africa has been identified by the Intergovernmental Panel on Climate Change (IPCC) as one of the continents most likely to be affected by climate change and the associated flooding disasters (IPCC 2022a; Schilling et al. 2020; Afuye et al. 2021a; Clarke et al. 2022). As a result, the African continent has experienced a high frequency of flood occurrences in recent decades (IPCC 2022b). A study pointed out that Africa and other regions must prioritise research-based education within their countries (Ngongalah et al. 2018). This can be achieved by building a solid foundation, increasing capacity, and providing sustainable training promoting research initiatives. Studies have shown that resource availability is crucial for research in developed and developing nations (Fedderke and Goldschmidt 2015; Huang et al. 2016). The results from this study will further advance the current body of knowledge in documenting and investigating African flood and South American cases. Consequently, evaluating the geographic distribution of studies on G_MFRA is essential for advancing our contemporary understanding of its global research trends for future development. This will aid the research community, institutions, and decision-makers in identifying key research hotspot areas by creating possible solutions to the adverse impacts of flood disasters.

Table 2 shows the top 20 most cited articles on geospatial and meteorological flood risk assessment (G_MFRA) studies. The distribution of top global articles per citation on G_MFRA can be found in journals related to the earth system, engineering, hydrology, environment, and water and land use, paying attention to this particular field of research. The assessment of these journals and publications in the top 20 productive articles, considering their development trends over time and total citations (TC), are shown in Table 2. For instance, Wiley Series in Systems Engineering and Management ranked first in G_MFRA studies, which produced (TC = 2295) with a powerful influence in the field, followed by Natural Hazards and Earth System Sciences (TC = 1460) and Hydrological Sciences Journal (TC = 1382) respectively. This implies that the distribution of publications in this field could be more prominent among the top journals, suggesting that it is dispersed across prominent journals and encompasses research erudition from various fields of study. Furthermore, it highlights the methodologies, indices, algorithms, remote sensing, and GIS-based techniques employed and their findings or gaps. Their conclusions were emphasised by the gaps in the literature and diverse flood models and methodologies such as the Global Circulation Model (GCM), multicriteria analysis, simple stochastic model, Monte Carlo framework, uncertainty analysis and holistic framework for flood risks, among others. This helped to improve our comprehension of flood risk assessment and the development of realistic scientific policies and practice management that contribute to reducing the risk of floods. Thus, these indices and models provide a first step in monitoring global change and broad spatiotemporal flood risk monitoring and flood indicators. The relationships between social changes and physical systems of human dynamics to the various economic effects of flood risk have only been partially researched using agent-based models (Sampson et al. 2015; Aerts et al. 2018). There is a knowledge vacuum in the assessments of flood risks since much research focuses on evaluating the existing techniques and the development of processes for estimating flood risk via uncertainty analysis of natural and human-induced elements (Haimes 2011; Kundzewicz et al. 2014; Kumar and Bhattacharjya 2020). Nevertheless, their results varied based on location, topography and climatic zone for different flood types based on methodologies employed and the distribution of flood occurrences (Table 2). Consequently, most studies show improvements in classifications and approaches to methodologies (De Moel et al. 2009; Yamazaki et al. 2019). For example, the hydrographic mapping and the innovative Sponge City Concept to absorb rain and prevent flooding thus outline the need for an optimised and well-structured data collection approach and the inclusion of socioeconomic factors (Merz et al. 2010; Zou et al. 2013; Chan et al. 2018). They also underscore the notion that changes in climatic conditions impact flood risk management practices. Therefore, the literature reviewed reveals the relationship between various extreme climate events and flood models, such as hydrological, meteorological, climatological, and geophysical models. These relationships significantly influence the natural environment. Thus, there still needs to be more research on the types of floods, methodologies, socioeconomic issues, and the notion that variations in the climate affect flood risk management practices. Thus, the nature of floods, socioeconomic factors, methodologies, and the idea that alterations in climate affect flood risk management strategies still need to be explored.

**Table 2:** Distribution of top 20 most-cited articles on geospatial and meteorological flood risk assessment studies

S/N	Title	Methodology	Findings/Gaps	Total citations	Journal	References
1.	Risk modelling, assessment, and management	Employed insights from engineering, economics, and decision sciences to provide a holistic framework for flood risk assessment and mitigation	The research delves into the complexities of risk modelling, emphasising the importance of probabilistic approaches and scenario analysis in capturing uncertainties. The findings highlight the significance of integrating stakeholder perspectives and fostering interdisciplinary collaboration to enhance risk management strategies.	2297	Wiley Series in Systems Engineering and Management	Haimes (2011)
2.	Assessment of economic flood damage	Employed a classification and analysis approach to elements at risk of flooding	Researchers noted a need for consistency in economic evaluation, emphasising systematic data collection and the definition of time and spatial boundaries for flood assessments.	1460	Natural Hazards and Earth System Sciences	Merz et al. (2010)
3.	Flood risk and climate change: global and regional perspectives	The study analysed literature from both the Intergovernmental Panel on Climate Change (IPCC) SREX report and recent assessments of changes in flood risk across continents including Africa, Asia, Central and South America, Europe, North America, Oceania, and Polar regions	The findings show how the specifics of these changes play a crucial role in shaping how flood characteristics respond to climate change. However, there is generally low confidence in projections of changes in river floods due to limited evidence and complex regional causes, leading to stronger uncertainties.	1382	Hydrological Sciences Journal	Kundzewicz et al. (2014)
4.	Flood risk assessment and associated uncertainty	Utilised a simple stochastic model through the Monte Carlo framework	The findings show two primary sources of uncertainties in flood risk assessments: natural and anthropogenic factors (aleatory uncertainty) and insufficient system knowledge (epistemic uncertainty).	706	Natural Hazards and Earth System Sciences	Apel et al. (2004)
5.	Global projections of river flood risk in a warmer world	An ensemble of seven high-resolution global climate projections based on Representative Concentration Pathways 8.5 to assess the changes in flood risk under specific warming levels of 1.50 C, 20 C and 40 C as compared to the preindustrial levels	The findings show that changes in flood risk vary widely across Asia, the U.S., and Europe, while they are not statistically significant for most of Africa and Oceania. At a 4 °C global warming scenario, countries representing more than 70% of the worldwide population and GDP will experience flood risk increases exceeding 500%.	668	Earth’s Future	Alfieri et al. (2017)
6.	Flood maps in Europe–methods, availability and use	This study used a review of current flood mapping practices in 29 European nations, highlighting the types of maps currently in circulation and their intended uses	The findings reveal that very few countries have created flood risk maps considering the consequences of flooding, with five nations needing comprehensive maps. However, other types of maps, like flood depth maps, are common in seven countries, while flood extent maps are prevalent in 23 nations. Despite these efforts, accurately assessing flood damage and incorporating indirect impacts like cultural and environmental damage remains a significant challenge.	638	Natural Hazards and Earth System Sciences	De Moel et al. (2009)
7.	Combining hazard, exposure, and social vulnerability to provide lessons for flood risk management	Conducted multicriteria analysis of various datasets to determine the Social Vulnerability Index (SVI)	The findings highlighted the importance of detailed household data for assessing social vulnerability per flood zone. They suggested effective flood risk management should consider socioeconomic status and spatial and societal characteristics. They predicted a 6% increase in the socially vulnerable population in the future.	590	Environmental science and policy	Koks et al. (2015)
8.	MERIT Hydro: A High‐Resolution Global Hydrography Map Based on the Latest Topography Dataset	Developed the MERIS DEM through multicriteria analysis of various datasets	Researchers indicated that creating a hydrologically correct DEM without integrating waterbody datasets was challenging. However, Open Street Map data proved effective for deriving stream channels narrower than the DEM pixel size.	568	Water Resources Research	Yamazaki et al. (2019)
9.	Debates: Perspectives on socio-hydrology: Capturing feedback between physical and social processes	Applied statistical empirical equations to capture dynamic data from both physical and social processes	The findings demonstrated that the new approach captured dynamic effects from both adaptation and levees, underscoring the influence of technology and societal memory on flood risk and management.	495	Water Resources Research	Di Baldassarre et al. (2015)
10.	A high‐resolution global flood hazard model	This study uses many recent developments in remote sensing and hydrology to propose a global flood hazard model framework for building a high-resolution (3 arc sec or ~ 90 m)	The findings show that the global flood hazard model faces challenges like topographical constraints at more minor scales and uncertainties, leading to underestimation of flood risks in coastal areas. Integrating a coastal component could alleviate this issue. The availability of new high-resolution terrain datasets like 1 arc sec SRTM and TanDEM-X Elevation12 DEM offers the potential for enhancing the model in the future.	495	Water Resources Research	Sampson et al. (2015)
11.	“Sponge City” in China: A breakthrough of planning and Flood Risk management in the urban context	Adopted the Low Impact Development (LID) approach for the “Sponge City” concept in China	Researchers found that the sponge city concept could mitigate urban flood risk in China by managing stormwater. However, it would not withstand severe storms exceeding the 1:30-year threshold.	483	Land Use Policy	Chan et al. (2018)
12.	Comparative flood damage model assessment: towards a European approach	Evaluated flood damage models through a qualitative and quantitative approach	The findings identified significant differences between various flood damage models in Europe and proposed an optimised pan-European framework. The study further noted that current models are needed to estimate damages to infrastructure accurately.	480	Natural Hazards and Earth System Sciences	Jongman et al. (2012)
13.	A multicriteria approach for flood risk mapping exemplified at the Mulde River, Germany	Developed a GIS-based multicriteria flood risk assessment using FloodCalc for spatial data processing	The findings suggested that modelling perceptions of flood risks and disaster risk reduction can improve communication and policy, highlighting the importance of spatial allocation of risks and mitigation measures.	473	Natural Hazards	Meyer et al. (2009)
14.	Integrating human behaviour dynamics into flood disaster risk assessment	Integrated flood-risk assessment models with behaviour adaptation dynamics using agent-based models (ABMs)	The findings highlighted the need for integrating human behaviours into flood risk assessment, pointing out the challenges of quantifying human behaviours’ influence on flood risks and mitigation measures.	473	Nature Climate Change	Aerts et al. (2018)
15.	Flood-risk mapping: contributions towards an enhanced assessment of extreme events and associated risks	The study proposes a new multifactorial method for estimating damage that considers building quality, pollution, and preventive actions to enhance the estimation of damage locally.	The findings show that the methods are used to develop comprehensive risk assessments and flood control strategies. The regionalisation approach enhances flood estimation for ungauged catchment areas and rare recurrence intervals, aiding in developing state-wide flood hazard maps and regional dam safety analyses.	439	Natural Hazards and Earth System Sciences	Büchele et al. (2006)
16.	A framework for global river flood risk assessments	Integrated climatic and socio-economic data in developing a new framework	The framework enabled effective prediction of global flood risk assessments by accurately estimating flood hazard and their consequences, highlighting the significant contribution of socioeconomic factors and climate change.	423	Hydrology and Earth System Sciences	Winsemius et al. (2013)
17.	Framework for economic pluvial flood risk assessment considering climate change effects and adaptation benefits	Included hazard and vulnerability analysis in a GIS-based risk model, focusing on climate change impact	The framework was used to emphasise climate change impacts on urban flood risks, showing that pipe enlargement is more cost-effective than local infiltration units for flood risk reduction.	411	Journal of Hydrology	Zhou et al. (2012)
18.	Use of Systematic, Palaeoflood and Historical Data for the Improvement of Flood Risk Estimation. Review of Scientific Methods	Combined systematic paleoflood and historical data with modern hydrological data	Researchers identified challenges in quantifying flood risks due to methodological complexity and called for integrating multidisciplinary approaches to enhance appropriate strategies in disaster management.	411	Natural Hazards	Benito et al. (2004)
19.	Comprehensive flood risk assessment based on set pair analysis-variable fuzzy sets model and fuzzy AHP	Combined the Set Pair Analysis and Variable Fuzzy Sets (SPA-VFS) in a hybrid approach	The findings show that the hybrid SPA-VFS model yielded practical flood risk assessments due to the organic integration of both models, highlighting the ease of computation and clarity.	409	Stochastic Environmental Research and Risk Assessment	Zou et al. (2013)
20.	Assessing flood risk at the global scale: model setup, results, and sensitivity	Utilised an integrated approach for global flood risk assessment, including climate change impact assessment	Researchers identified the need for integrating risk assessment tools with socio-economic tools for adaptation, stressing the inclusion of indirect intangible value in economic analyses.	390	Environmental research letters	Ward et al. (2013)

Hence, considering the empirical knowledge and evidence-based methodologies that explain the inputs of institutional strengthening of response strategies and adaptive capacity to flood risks has yet to draw much attention (Huntjens et al. 2012; Doswald et al. 2014; Yu et al. 2021). Therefore, to fill the gap in the literature, the present study assessed available published articles on geospatial and meteorological flood risk assessment (G_MFRA) studies to highlight the key themes, methodologies, global trends in flood risk research and practice and emerging directions. SDG_11_ (Sustainable Cities and Communities) and SDG13 (Climate Action) were highlighted in line with G_MFRA to corroborate further the findings presented in the study. Therefore, the challenges identified in the body of extant literature about technical expertise, complex communication networks, and resource constraints are associated with the application gaps in the study methodologies. The study emphasises the need for a multidisciplinary approach, encompassing environmental and socioeconomic factors and utilising cutting-edge algorithms and technology. This comprehensive strategy is crucial for establishing an integrated and sustainable framework for flood risk assessment.

**Fig. 7:**
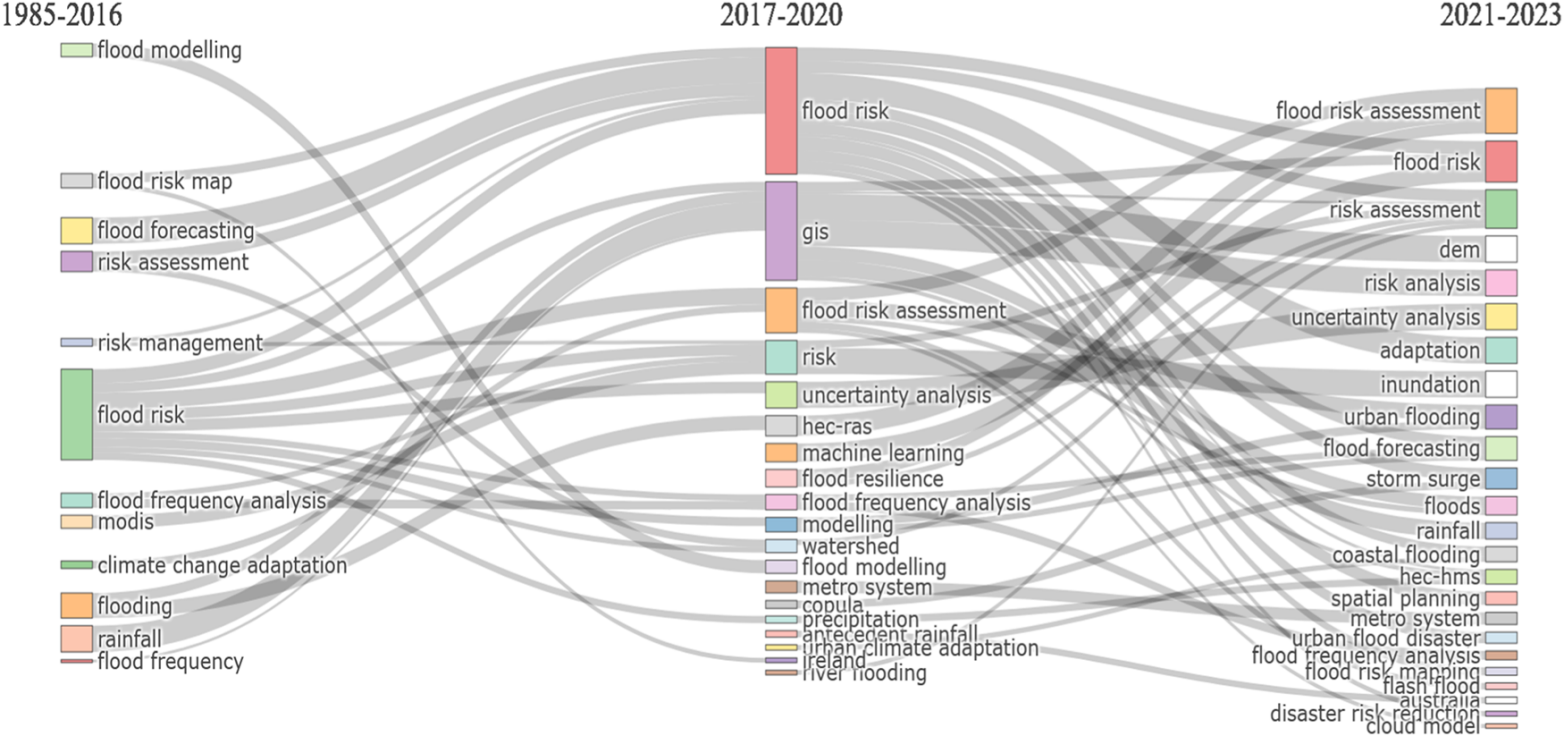
Thematic evolution of author’s keywords on G_MFRA studies

Figure 7 illustrates how the conceptual progression of the author’s themes in G_MFRA was used to determine the thematic advancement, research clusters, and origin based on the occurrence of crucial phrases in the field. The height of the bars represents the frequency of publications often used to comprehend and visualise how the thematic evolution of keywords changes over time (Lei and Xu 2020; Casado‐Aranda et al. 2023). The thematic evolution, research clusters, and progress represent how key themes appeared in the selected authors’ keywords over time. The thematic evolution of the author’s keywords revealed the dominating stable themes clustered into three research timeframes using a structured scheme between 1985–2016, 2017–2020 and 2021–2023, as shown in Fig. 7. The results show that the most stable author’s themes with significant appearance were flood risk in 1985–2016, accompanied by flooding and rainfall and flood forecasting, which are interconnected to several methodologies used in the research domain. These methodologies include GIS, uncertainty analysis, machine learning, flood frequency analysis and Hydrologic Engineering Center - River Analysis System (HEC-RAS), which evolved into coastal flood modelling and became prominent in 2017–2020. The last segment of 2021–2023 shows that flood risk assessment, flood risk and risk assessment remain the commonly used keyword themes in G_MFRA studies in recent decades. In addition, the last segment added the hydrologic modelling system (HEC-HMS), spatial planning, metro system and cloud model, among others. It is worth noting that flood risk and associated themes, such as risk assessment and management, have been utilised in the literature. However, their usage in flood risk assessment research has been minimal. This draws attention to the gaps in research on flood risk management and the application of geographical and meteorological data and methodologies. The increasing frequency of floods and inundation events in developing and low-income countries that are most vulnerable to inadequate response and recovery measures have not been the focus of many studies on G_MFRA (Tayyab et al. 2021; Membele et al. 2022; Nguyen et al. 2023). The importance of promoting more robust and sustainable methods of coping with flood disasters and advancing disaster risk reduction (DRR) policies globally is emphasised in the Sendai Framework for Disaster Risk Reduction 2015–2030 (Busayo et al. 2020; Vitale 2023). This aligns with the Sustainable Development Goals of shifting from risk-based to resilience-based flood management and flood disaster risk control through ecosystem-based adaptation. Furthermore, socioeconomic factors, including population density, infrastructure, vulnerability, topography, land use, soil type and community resilience, might not be fully included in G_MFRA research. Most studies have not considered these aspects, resulting in insufficient mitigation techniques and incomplete flood risk assessments. Therefore, the thematic evolution of the author’s keywords further highlights the scientific progress and research gaps in applying geospatial and meteorological flood risk assessment studies.

## Potential Flood Adaptive Measures, Disaster Mitigation Strategies and Policy Implications

Proactive measures and effective strategies can mitigate the effects of natural disasters such as floods and their associated societal impacts. Monitoring systems, flood frequency analysis, AHP, risk analysis, risk models, HEC-RAS, GIS, and other predictive technologies have been used in conjunction with response procedures and are essential components of flood disaster mitigation strategies (Gacu et al. 2022; Majeed et al. 2023). The possibilities of predictive technology have yet to be thoroughly explored in geospatial and meteorological flood risk assessment (Ahmad et al. 2013; Emerton et al. 2016; Zennaro et al. 2021; Kumar et al. 2023). Advances in space-based technologies can enhance socio-environmental systems and prediction capabilities to precisely forecast the occurrence, severity, and path of floods that create uncertainties. This underscores the importance of geospatial and meteorological applications in assessing flood disaster risks. This study shows that coordinated research efforts and policy response became crucial compared to other continents due to the growing consequence of flood risk across North America, Europe, Asia, and Australia. The findings demonstrate that developing continents like Africa and South America lack the essential resources to cope with flood risks, underscoring the significance of adaptive governance in these regions. Institutional strengthening of response strategies and adaptive capacity to flood risk in these regions necessitates integrating indigenous knowledge and appropriate policies. In geospatial and meteorological applications for flood risk assessment, bridging the gap between research and policy is crucial for informed decision-making. This study shows that geospatial and meteorological approaches are pivotal in managing flood disasters exacerbated by natural and human-induced activities. Furthermore, this study advocates for further research to develop innovative flood techniques and models with the potential to influence global decision-making in the G_MFRA domain. Addressing the identified research gaps can bolster flood risk assessment and management efforts by advancing their applicability in the field.

### Global Trends in Flood Risk Research and Practice

Floods are one of the most significant risks to communities globally. Thus, efforts to lessen their effects must continue in research and practice. The analysis of global research trends in flood risk and practice highlights the key themes, methodologies, and emerging directions. Appraising these available articles on G_MFRA studies provides advances in research hotspots and areas of flood risks, enabling global policy interventions using bibliometrics of academic literature. The study identified key trends and themes, current challenges, and opportunities for advancing flood risk management worldwide by synthesising literature from published articles in G_MFRA. The following topics might be relevant to more research and practice in the field. This includes further research on remote sensing applications for flood monitoring and mapping, integration of meteorological data with hydrological models, climate change impacts on flood risk dynamics, decision support systems for flood mitigation and emergency response, community-based participatory approaches to flood risk management and case studies and best practices from different regions and climatic zones etc. Therefore, the evolving landscape of flood risk research and practice reflects the growing recognition of floods as a global challenge that demands interdisciplinary solutions. This study provides a comprehensive overview of the current state of research on geospatial mapping and meteorological flood risk assessment. This offers valuable insights for scholars, practitioners, and policymakers engaged in disaster management and climate resilience efforts. Developing an efficient range of data and methodologies would further improve our understanding of flood disasters, and leveraging technological advances and interdisciplinary collaborations can contribute to building more resilient communities globally.

### Limitations and Future Prospects

Communication challenges translating complex geospatial and meteorological data into actionable insights for policymakers and the public can be challenging. It is crucial to communicate flood disasters and mitigation techniques effectively. Nevertheless, it requires specialised knowledge in both the technical and communication domains. Four evolutionary paths have been identified: integrating the big data approach, GIS-based models to urban social vulnerability, machine learning-based early warning systems and multidisciplinary approaches to flood risk. Continued research on G_MFRA and key technological advancements in the current research trend will address these limitations and improve the accuracy based on methodologies and effectiveness of flood risk assessments and mitigation strategies. Despite these limitations, geospatial mapping and meteorological flood risk assessment remain invaluable tools for understanding and mitigating flood events and associated shear risks. However, this research will help governments and organisations implement a multi-integrated theoretical framework that includes preparedness and response techniques for disaster mitigation and adaptive flood control. This study navigates the knowledge base surrounding the G_MFRA research, which offers a basis for future research development in this specialised field. It is important to stress that this study was streamlined and focused on significant studies that explored the core areas of G_MFRA research. This is crucial to identifying areas of focus in this niche area. Therefore, as new research evolves, it is anticipated that the latest empirical studies and viable solutions will provide new insights on broader issues about flood risk and sustainable flood management. The scope of the study is limited by its integration of articles indexed in the WOS and Scopus databases. Future studies may use other databases to offer more prospective research areas and advancements.

## Conclusion

This study appraised studies on G_MFRA to reveal the evolutionary trend and advances in global research hotspots and a better understanding of dominant themes using bibliometric analysis of published articles between 1985 and 2023. The assessment and approach outlined in this study contribute to deepening our comprehension of the intellectual landscape of global research hotspots, the distribution of top global articles, collaboration networks, the evolution of keyword co-occurrence, trending topics, and institutional affiliations related to G_MFRA studies. The findings from this review show that “flood risk and “risk assessment” are prominent in all keywords with the utmost relevance within disaster science. China emerged as a leading contributor to G_MFRA research, demonstrating significant influence through high citation rates, research output, funding, and collaborations with various research centres. Other notable contributors include Germany, the Netherlands, the United Kingdom, and Italy. Collaboration among these countries across continents aided the advancements in G_MFRA, which are mainly driven by technological innovation and financial capabilities in Asia, Europe, North America, and Australia. The study shows that research from Africa and South America has been limited, likely due to insufficient funding and collaboration networks. The study calls for increased attention to underrepresented regions in flood risk research to address geographical biases and promote global research coverage.

The study explores advancements in geospatial and meteorological flood risk assessment (G_MFRA) studies, emphasising the need to evaluate existing research to identify gaps and areas for improvement, particularly in underrepresented regions. The assessed keywords highlight the need for more research and emphasise the importance of addressing gaps in understanding flood impacts on communities and improving adaptive flood mitigation systems. The study underscores the importance of Sustainable Development Goals SDG_11_ and SDG_13_ in addressing flood-related climate change impacts and urban resilience. It also highlights the necessity for further research on flood estimation uncertainties and the correlation between floods and climate change-induced human impacts. The findings suggest the need for more comprehensive research utilising advanced geospatial techniques to enhance flood disaster risk management and ecosystem-based adaptation in alignment with the SDGs. The study suggests further integration of flood risk reduction initiatives such as flood risk management strategies (FRMSs), Flood Resilience Enhancement and Management (FREEMAN), the National Flood Insurance Program (NFIP) and the first African Risk Capacity (ARC) for flood Risk Insurance product as flood monitoring tools. This will provide robust, timely financing for flood-related emergencies and the use of different datasets to monitor flood-induced changes at both global and regional scales.

## Data Availability

Data used in this study is available on request.
